# Evaluation of exclusive enteral nutrition and corticosteroid induction treatment in new-onset moderate-to-severe luminal paediatric Crohn’s disease

**DOI:** 10.1007/s00431-022-04496-7

**Published:** 2022-06-08

**Authors:** Maria M. E. Jongsma, Stephanie A. Vuijk, Martinus A. Cozijnsen, Merel van Pieterson, Obbe F. Norbruis, Michael Groeneweg, Victorien M. Wolters, Herbert M. van Wering, Iva Hojsak, Kaija-Leena Kolho, Michiel P. van Wijk, Sarah T. A. Teklenburg-Roord, Tim G. J. de Meij, Johanna C. Escher, Lissy de Ridder

**Affiliations:** 1 Department of Pediatric Gastroenterology, Erasmus Medical Centre-Sophia Children’s Hospital, Room SP-2430, P.O. Box 2040, 3000 Rotterdam, CA Netherlands; 2 Department of Pediatric Gastroenterology, Isala Hospital, Zwolle, Netherlands; 3 Department of Pediatric Gastroenterology, Maasstad Hospital, Rotterdam, Netherlands; 4 Department of Pediatric Gastroenterology, Utrecht Medical Centre-Wilhelmina Children’s Hospital, Utrecht, Netherlands; 5 Department of Pediatric Gastroenterology, Amphia Hospital, Breda, Netherlands; 6 Referral Centre for Pediatric Gastroenterology and Nutrition, Children’s Hospital Zagreb, University of Zagreb Medical School, Zagreb, Croatia; 7 Tampere University Hospital and University of Tampere, Tampere, Finland; 8 Children’s Hospital, University of Helsinki, Helsinki, Finland; 9 Department of Pediatric Gastroenterology, Amsterdam UMC-Emma Children’s Hospital, VU University, Amsterdam, Netherlands

**Keywords:** Mucosal healing, Endoscopic remission, Inflammatory bowel disease, Child, Adolescent, Crohn’s disease

## Abstract

To induce remission in luminal paediatric Crohn’s disease (CD), the ESPGHAN/ECCO guideline recommends treatment with exclusive enteral nutrition (EEN) or oral corticosteroids. In newly diagnosed moderate-to-severe paediatric CD patients, we determined the proportion of patients in which EEN or corticosteroids induced remission and maintained remission on azathioprine monotherapy. We included patients from the “TISKids” study assigned to the conventional treatment arm. Patients were aged 3–17 years and had new-onset, untreated luminal CD with weighted paediatric CD activity index (wPCDAI) > 40. Induction treatment consisted of EEN or oral corticosteroids; all received azathioprine maintenance treatment from start of treatment. The primary outcome of this study was endoscopic remission defined as a SES-CD score < 3 without treatment escalation at week 10. Secondary outcomes included proportion of patients without treatment escalation at week 52. In total, 27/47 patients received EEN and 20/47 corticosteroids. At baseline, patient demographics and several inflammation parameters were similar between the two treatment groups. At 10 weeks, clinical remission rates were 7/23 (30%) for EEN and 7/19 (37%) for corticosteroids (*p* = 0.661). Twenty-nine of 47 consented to endoscopy at 10 weeks, showing endoscopic remission rates without treatment escalation in 2/16 (13%) of EEN-treated patients and in 1/13 (8%) of corticosteroid-treated patients (*p* = 1.00). At week 52, 23/27 (85%) EEN-treated patients received treatment escalation (median 14 weeks) and 13/20 (65%) corticosteroid-treated patients (median 27 weeks), *p* = 0.070.

*Conclusion*: In children with moderate-to-severe newly diagnosed CD, induction treatment with EEN or CS regularly is insufficient to achieve endoscopic remission without treatment escalation at week 10. *Trial registration number*: NCT02517684

**Table:** 

**What is Known:** *• Endoscopic remission is associated with a low risk of disease progression.* *• FL-IFX was superior to conventional treatment in achieving and maintaining remission in paediatric patients with moderate-to-severe CD the first year from diagnosis.*	
**What is New:** *• In children with newly diagnosed moderate-to-severe CD, clinical remission rates and endoscopic remission rates without treatment escalation at week 10 were 30% and 13% after EEN and 37% and 8% after corticosteroid induction treatment.* *• The current treatment target was often not achieved by either EEN or corticosteroid induction treatment after bridging to azathioprine.*

**Supplementary Information:**

The online version contains supplementary material available at 10.1007/s00431-022-04496-7.

## Introduction

Crohn’s disease (CD) is a chronic inflammatory disease which may affect the entire gastrointestinal tract [1]. Up to one in ten patients is diagnosed during childhood [2]. In the last decade, the treatment goal for paediatric CD patients has changed from control of symptoms to a “treat-to-target” strategy, aiming at endoscopic remission with a higher chance of significantly improving the disease course. Achievement of endoscopic remission is associated with a low risk of disease progression [3]. Timely and individualized interventions are crucial to reduce inflammation and thus prevent irreversible bowel damage and complications [4]. For children with uncomplicated luminal CD, the start of conventional treatment, involving exclusive enteral nutrition (EEN) or oral corticosteroids to induce remission, combined with azathioprine (AZA) to maintain remission, is recommended according to the current European Crohn’s and Colitis Organisation (ECCO)/European Society for Paediatric Gastroenterology, Hepatology and Nutrition (ESPGHAN) guideline (2020) [5].

In these patients, EEN and oral corticosteroids have proven to be equally effective in inducing clinical remission [6]. While the mechanism of action of EEN is not yet fully understood, EEN is known to have a direct anti-inflammatory effect on the intestine in patients with CD [7]. However, the 6–8 week period of complete liquid formula, with no other food or drinks allowed, is hard to comply with. On the other hand, oral corticosteroid treatment has a high risk of side effects, such as increased infection rate, Cushingoid appearance, bone demineralization, and growth retardation [8].

In the TISKids randomized controlled trial, it has been shown that in paediatric patients with newly diagnosed moderate-to-severe CD, induction treatment with first-line infliximab (FL-IFX) was superior to conventional treatment in achieving and maintaining remission and linear growth in the first year from diagnosis [9].

Still, EEN and oral corticosteroids are widely used to induce remission in paediatric CD. Therefore, the aim of this study is to compare clinical, biochemical, and endoscopic response and remission achieved by EEN or corticosteroids in the population of newly diagnosed moderate-to-severe luminal paediatric CD patients. Moreover, it will be assessed whether AZA maintenance therapy is capable of maintaining remission.

## Methods

### Procedures

This study was a secondary analysis of the TISKids study [9]; patients receiving conventional treatment per protocol were included. The choice for either EEN or corticosteroids within the conventional treatment group was up to the patient and parents, in accordance with the treating physician. Details of the TISKids study design and protocol were previously published [10]. Conventional treatment consisted of induction treatment with EEN (polymeric feeding for 6–8 weeks after which normal diet was gradually reintroduced within 2–3 weeks) or oral corticosteroids (1 mg/kg prednisolone daily with a maximum of 40 mg for 4 weeks, followed by tapering down 5 mg per week until stop) combined with AZA as maintenance treatment (2–3 mg/kg, once daily) which was started simultaneously with the induction treatment. In case of thiopurine methyl transferase (TPMT) heterozygosity, AZA dosing was halved. AZA metabolites (6-thioguanine nucleotides and 6-methylmercaptopurine) were measured as part of clinical care. 6-TGN levels below < 230 pmol/8 × 10^8^ RBC were considered insufficient [11]. It was advised to check AZA metabolites in case of loss of response to AZA monotherapy. In all centers, the same protocol was followed and compliance of patients was assessed by the dietician and treating physicians as part of regular inflammatory bowel disease (IBD) care.

Changes in treatment could be made according to the physician’s discretion in patients without response, loss of response, or intolerance to treatment. Additional CD-related therapy included initiation of IFX, any course of EEN, and any course of corticosteroids that was additional to the standard treatment described in the previous section.

Data were collected prior to start of induction therapy, at weeks 6, 10, 14, 22, and 52. At each visit, weighted paediatric CD activity index (wPCDAI) was determined [12]; blood was obtained for routine laboratory analysis. Endoscopy (ileocolonoscopy) was performed prior to start of treatment at week 10, and optionally at week 52. During endoscopy, the Simple Endoscopic Score for Crohn’s Disease (SES-CD) was used to evaluate endoscopic remission [13], which was defined as SES-CD score < 3. Endoscopic response was defined as a reduction in SES-CD score by 50%. A single reader, blinded for both assigned treatment and time point, evaluated and rescored all endoscopic still images available by using the physician global assessment endoscopy score [14], to check inter-observer variability between paediatric gastroenterologists (*r* = 0.689, *p* < 0.001). Faecal samples were collected for faecal calprotectin (fcal) level measurement prior to start of treatment, at week 10, and at week 52. Fcal levels were assessed in the Erasmus Medical Centre with ELISA (CALPRO assay). When fcal samples were missing, fcal levels determined in the local hospital at this time point were used, which accounted for 13% of all samples. A fcal level < 100 µg/g was defined as biochemical remission [15]. Standard deviation scores (SDS) adjusted for sex and age were used to evaluate linear growth. The SDS were calculated based on the Dutch national reference standards for all patients included in the Netherlands and the World Health Organization (WHO) growth reference standards for all patients included in other countries. Target height and target height SDS were calculated [16]. The Growth Analyser Research Calculation Tool was used to calculate the SDS based on these references [17].

### Outcomes

#### Primary outcome

The primary outcome of this study was endoscopic remission defined as a SES-CD score < 3 without treatment escalation at week 10.

#### Secondary outcomes

Secondary outcomes included time-to-treatment escalation from start of induction and clinical disease activity scores over time. At week 6, clinical remission rate (wPCDAI < 12.5) was assessed. At week 10, clinical remission rate and fcal levels were assessed. At week 14, treatment success was assessed, which was defined as clinical remission without treatment escalation. At week 52, the following outcomes were assessed: (1) cumulative corticosteroid use, (2) need for treatment escalation, (3) linear growth, (4) clinical remission rate, (5) endoscopic remission rate, (6) fcal level. In addition, the association between patients’ characteristics at baseline and treatment success at week 14 as well as IFX use at week 52 was evaluated.

### Statistical analysis

Continuous variables were presented as medians and interquartile ranges (IQR) and compared with the Mann–Whitney *U* test. Categorical variables were presented as absolute frequencies and percentages and compared by the *X*^2^ test or the Fisher exact test. SES-CD scores with a missing ileum sub-score due to the endoscopist’s failure to intubate the terminal ileum were included in the analysis to evaluate endoscopic remission. Patients were analyzed according to the treatment group (EEN or prednisolone) they were initially assigned to. Complete clinical disease activity scores were not available for all patients at every visit. Analysis of clinical disease activity was performed on complete scores at each visit. The median fcal levels and SES-CD scores were subject to right censoring. To correct for this, medians of fcal levels and SES-CD scores were calculated using the Kaplan–Meier method, and treatment groups for these outcomes were compared using the log rank test. The time to treatment escalation outcomes were analysed using the Kaplan–Meier method. A paired analysis was performed for the linear growth. A logistic regression analysis was performed to analyse the association between baseline patients’ characteristics with moderate or severe disease, ESR, CRP, and treatment at week 14 and 52.

All analyses were performed based on a significance level of 0.05. Calculations were performed using IBM SPSS Statistics 24.0 (IBM Corp, Armonk, NY). The TISKids trial is registered in ClinicalTrials.gov, number NCT02517684.

### Ethical considerations

Medical-ethical approval was obtained for each site.

## Results

**Fig. 1:**
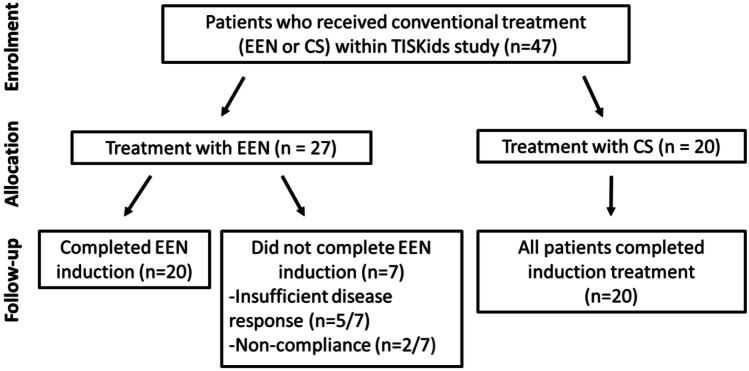
Flow diagram of conventionally treated patients of the TISKids study. Three patients who were treated with CS, completed induction treatment with CS but started IFX as treatment escalation while tapering prednisolone. EEN, exclusive enteral nutrition; CS, corticosteroids

Forty-seven patients received conventional induction treatment per protocol. To induce remission after diagnosis, 27/47 (57%) patients were treated with EEN, while 20/47 patients (43%) were treated with oral corticosteroids. Patients’ characteristics at baseline were, except for fcal levels, similar between the two treatment groups. Fcal levels were significantly higher in the EEN group (median 1197 (1033–1661)) compared to those in the corticosteroid group (592 (555–1133), *p* = 0.01) (Table 1). Twenty out of 27 (74%) EEN-treated patients completed the remission induction treatment. EEN was prematurely ended due to insufficient disease response in 5/7 patients and due to low compliance in 2/7 patients (Fig. 1). These patients all received step-up treatment with corticosteroids. Of the patients who completed EEN treatment, the median duration of EEN treatment was 43 days (IQR 42.0–44.0). All 20 patients with corticosteroids completed the induction treatment of median 30 days (IQR 28–34), whereafter the dose was tapered.

**Table 1:** Baseline characteristics of patients treated with exclusive enteral nutrition versus corticosteroids

		**EEN (*n* = 27)**	**Corticosteroids (*n* = 20)**	***p* value**
**Age at diagnosis, years**		14.5 (12.4–16.5)	13.8 (11.6–15.6)	0.282
**Male sex**		16 (59%)	10 (50%)	0.528
**Height, cm**		164.3 (149.2–175.0)	157.4 (143.5–16.6)	0.169
**Weight, kg**		48.5 (33.8– 56.3)	41.8 (31.1–53.9)	0.240
**SDS height for age**		–0.49 (–1.08 to 0.30)	–0.80 (–1.10 to –0.11)	0.533
**wPCDAI**		60.0 (48.8–67.8)	53.8 (45.6–81.3)	0.909
**CRP, mg/L**		34.5 (24.7–65.9)	39.5 (20.5–69.0)	0.921
**ESR, mm/h**		27.0 (17.0–47.5)	34.0 (22.5–71.0)	0.091
SES-CD*		15 (6–22)	19 (6–28)	0.275
**Leukocytes, 10**^**9**^**/L**		8.9 (6.8–11.7)	9.1 (6.2–11.8)	0.982
**Faecal calprotectin, µg/g**		1197 (1033–1661)	592 (555–1133)	0.014
Perianal disease**		5 (19%)	4 (20%)	0.898
**Paris classification**				
**Age at diagnosis**	< 10 years	4 (15%)	3 (15%)	0.757
	10–17 years	20 (74%)	16 (80%)	
	17–40 years	3 (11%)	1 (5%)	
**Disease location**	L1	6 (22%)	5 (25%)	0.755
	L2	6 (22%)	6 (35%)	
	L3	15 (56%)	9 (45%)	
	Isolated L4	-	-	
**Upper disease location**	No upper GI	14 (52%)	11 (55%)	0.942
	L4a	12 (44%)	8 (45%)	
	L4b	1 (4%)	1 (5%)	
**Disease behaviour**	B1	25 (93%)	16 (80%)	0.201
	B2	2 (7%)	4 (20%)	
	B3	-	-	
	B2B3	-	-	
**Growth delay**		1 (4%)	1 (5%)	0.828
**Time between diagnostic endoscopy** **and start of treatment, days**		7 (1–13)	8 (3–17)	0.266

Data are *n* (%) or median (IQR)**.** *wPCDAI*, weighted paediatric Crohn’s disease activity index; *CRP,* C-reactive protein; *ESR*, erythrocyte sedimentation rate; *SES-CD*, Simple Endoscopic Score for Crohn’s Disease

^*^Terminal ileum was not intubated in 6/20 (30%) of the patients treated with corticosteroids and in 6/27 (22%) of the patients treated with EEN (*p* = 0.545)

^**^Perianal disease comprised inactive fistula, skin tags, or anal fissures

### Efficacy of induction treatment

Twenty-nine out of 47 (62%) patients consented to the endoscopic evaluation per protocol at 10 weeks. Thereof, 5/16 (31%) EEN-treated patients and 4/13 (31%) of the corticosteroid-treated patients showed endoscopic treatment response (*p* = 0.978). Endoscopic remission without treatment escalation was found in 2/16 (13%) EEN-treated patients and 1/13 (8%) corticosteroid-treated patients (*p* = 1.00). Moreover, one out of 16 patients (6%) in the EEN group did achieve endoscopic remission, but received treatment escalation before 10 weeks. Remarkably, in the group of patients that underwent endoscopy, wPCDAI was significantly lower than in patients in whom repeated endoscopy was not performed (Supplemental Table 1).

### Clinical remission rates

At week 6, 11/25 patients (44%) treated with EEN were in clinical remission, compared to 10/18 patients (56%) patients treated with corticosteroids (*p* = 0.455). A decrease of the clinical remission rate was seen at week 10. At week 10, 7/23 patients (30%) EEN and 7/19 (37%) patients treated with corticosteroids were in clinical remission (*p* = 0.661). In line with the endoscopic findings, in both groups, only a minority of patients had fcal levels below 100 µg/g (EEN: 3/21 (14%) vs. corticosteroids: 2/16 (13%), *p* = 0.875) at week 10. At week 14, five out of 25 (20%) of the EEN-treated patients and 9/19 (47%) of the corticosteroid-treated patients were in clinical remission without treatment escalation after induction treatment (*p* = 0.054). One of these 14 patients switched to methotrexate after 4 weeks due to intolerance of AZA.

### Treatment course

**Fig. 2:**
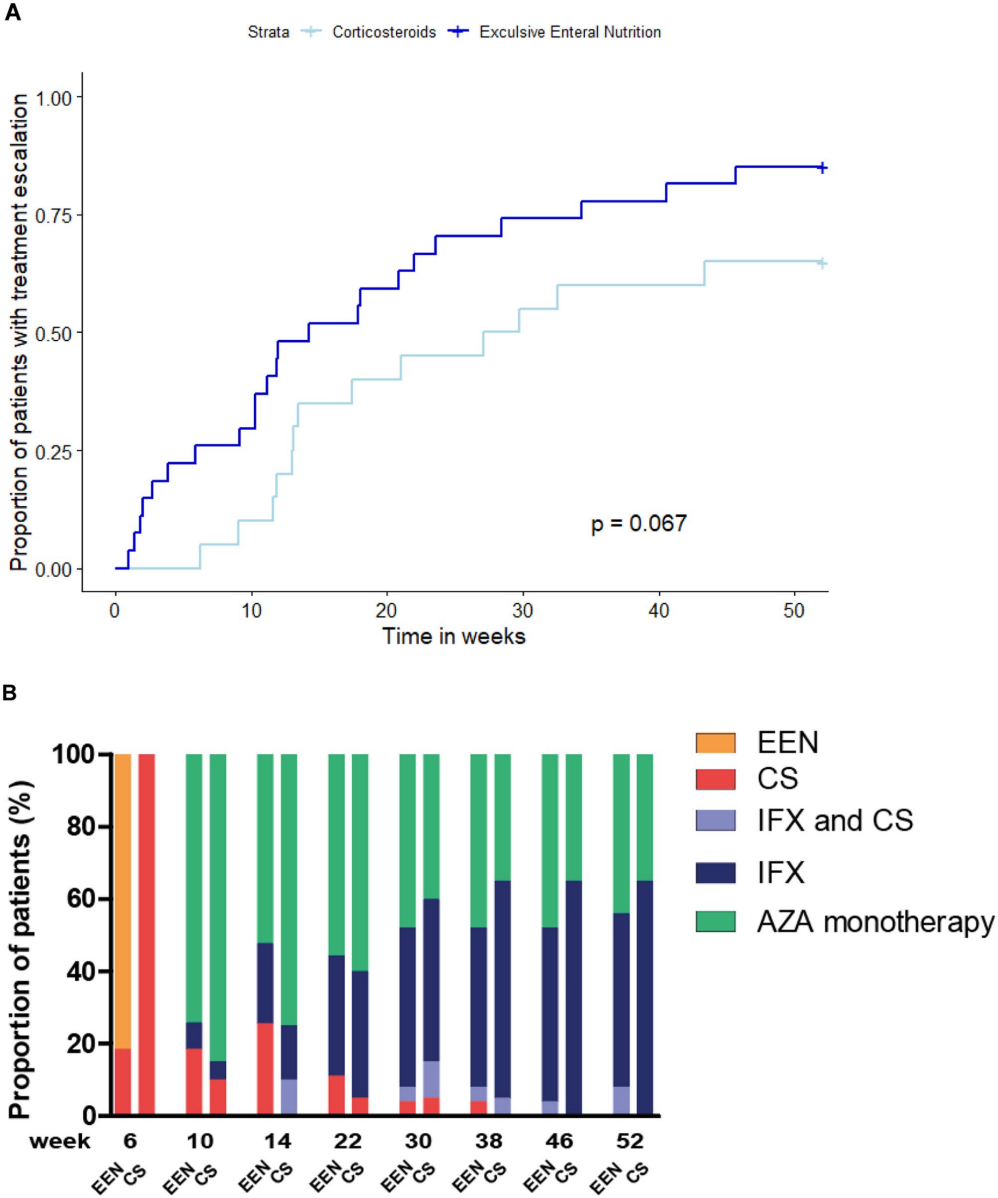
**A** Kaplan-Meier estimates of the time to treatment escalation after start of therapy. Any additional CD-related therapy or surgery during the 52 weeks was considered treatment escalation. **B** Proportion of patients receiving each treatment option from 6 weeks onwards, depicted per treatment group. EEN, exclusive enteral nutrition; CS, corticosteroids’; IFX, infliximab; AZA, azathioprine

Overall, if the primary induction treatment was taken into account the total number of days on corticosteroids was median 78 (71–103) days: for the corticosteroid-treated patients, which was significantly more than that in the EEN group (median 54 (0–70) days; *p* < 0.001). Twenty-three out of 27 (85%) EEN-treated patients received treatment escalation after a median of 14 weeks and 13/20 (65%) corticosteroid treated patients after a median of 27 weeks, *p* = 0.067 (Fig. 2a). In addition, a similar percentage of patients used IFX at week 52, 16/27 (59%) of EEN-treated patients were escalated to IFX at week 52, while this yielded 13/20 (65%) of the patients treated with corticosteroids (*p* = 0.689) (Fig. 2b).

### One-year follow-up

At week 52, no significant differences were found between treatment groups in the proportion of patients in clinical, biochemical, and endoscopic remission without treatment escalation at week 52 (Table 2). Out of the 14 patients who achieved treatment success at week 14, 2/5 (40%) of the EEN-treated patients and 4/9 (44%) of the corticosteroid treated patients were in clinical remission without treatment escalation at 52 weeks on AZA monotherapy (*p* = 0.872). In 11/14 (79%) patients, AZA metabolites were assessed. Nine out of 11 (81%) patients had adequate AZA trough levels. One of the 2 patients without adequate 6-TGN levels was not in clinical remission at 52 weeks without treatment escalation.

**Table 2:** Findings at week 52 per treatment group

	**EEN (*n* = 27)**	**Corticosteroids (*n* = 20)**	***p*-value**
wPCDAI, median (IQR)	4 (0–14)	11 (0–28)	0.158
Clinical remission (wPCDAI < 12.5)	14/23 (61%)	9/18 (50%)	0.486
Endoscopic remission, *n* (%)*	2/6 (33%)	3/7 (43%)	0.587
SES-CD, median (IQR) **	3 (0–6)	4 (1–7)	0.838
Fcal < 100 µg/g, *n* (%)	5/17 (29%)	3/13 (23%)	0.697
SDS height for age, median (IQR)	–.64(–.97 to 0.10)	–.86 (–1.3 to 0.19)	0.688

Clinical remission is defined as a wPCDAI < 12.5. Endoscopic remission was defined as a SES-CD < 3

*wPCDAI *weighted paediatric Crohn’s disease activity index (range 0–125), *SES-CD* Simple Endoscopic Score for Crohn’s Disease (range 0–60), *Fcal*, faecal calprotectin level

^*^6 EEN patients and 7 prednisolone-treated patients consented for endoscopy at week 52. Baseline characteristics of these patients are similar

^**^Terminal ileum was not intubated in 1/7 (14%) of the patients treated with corticosteroids and in 2/6 (33%) of the patients treated with EEN (*p* = 0.559)

Surprisingly, SDS height for age decreased significantly in EEN-treated patients (− 0.49 to − 0.64 (*p* = 0.016)), while SDS height for age was stable in corticosteroid-treated patients (− 0.80 at baseline to − 0.86 (*p* = 0.615)) (Table 2). However, in the subgroup of patients who were able to complete EEN induction treatment (*n* = 20), SDS height for age numerically decreased after 52 weeks (− 0.51 at baseline to − 0.65 after 52 weeks although this difference was not statistically significant (*p* = 0.086)). At 52 weeks, SDS height for age was not significantly different between treatment groups (*p* = 0.688). During the first year after diagnosis, in both treatment groups, one patient underwent an ileocecal resection. Three EEN-treated patients received surgery due to a perianal abscess.

### Patients’ characteristics at diagnosis associating with clinical disease outcomes

In a univariate analysis, moderate clinical disease scores significantly increased the odds of having treatment success at week 14 (OR 5.50 [1.26–23.94], *p* = 0.023) compared to severe clinical disease scores at baseline (Table 3A). In multivariable regression analysis, patients treated with corticosteroids had significantly higher odds of having treatment success at week 14 (OR 5.49 [1.13–26.62], *p* = 0.035) compared to EEN, irrespective of having moderate or severe clinical disease scores at diagnosis (Table 3B). None of the patient characteristics at baseline was significantly associated with IFX use within 1 year (Table 3A and 3C).

**Table 3:** Association of patients’ characteristics with sustained remission at week 14 and IFX use at week 52. **A**: Univariate analysis of associations with patients’ characteristics and treatment success at week 14 and IFX use at week 52. **B**: Multivariate analysis for associations with treatment success at week 14. **C**: Multivariable analysis for associations with IFX use at week 52. For categorical covariates, the last category (severe disease, EEN treatment) was the reference category. *Moderate disease*, wPCDAI 40–57.5; *Severe disease*, wPCDAI > 57.5; *CRP*, C-reactive protein; *ESR*, erythrocyte sedimentation rate; *Fcal*, faecal calprotectin level

3A				
	Treatment success at week 14 Odds ratio [95% CI]	*p*-value	IFX use at week 52 Odds ratio [95% CI]	*p*-value
Corticosteroid or EEN treatment	3.60 [0.95–13.62]	0.059	1.28 [0.39–4.23]	0.689
Disease location at diagnosis	1.44 (0.63–3.29)	0.386	0.87 [0.42–1.80]	0.708
Moderate or severe disease at diagnosis	5.50 [1.26 – 23.94]	**0.023**	0.52 [0.16 – 1.71]	0.280
SES-CD at diagnosis	1.01 [0.94–1.09]	0.784	1.01 [0.94–1.08]	0.821
Albumin at diagnosis	1.02 [0.96–1.08]	0.571	0.94 [0.85–1.04]	0.252
ESR at diagnosis	0.97 [0.93 –1.00]	**0.049**	1.01 [0.98–1.04]	0.510
CRP at diagnosis	0.98 [0.96 – 1.01]	0.113	1.01 [0.99 – 1.03]	0.377
Fcal at diagnosis	1.00 [1.00–1.00]	0.296	1.00 [1.00–1.00]	0.659

3B		
	Odds ratio [95% CI]	*p*-value
Moderate or severe disease at diagnosis	2.69 [0.46 – 15.65]	0.271
ESR at diagnosis	0.97 [0.94 –1.01]	0.110
CRP at diagnosis	0.99 [0.96 – 1.02]	0.548
Corticosteroid or EEN treatment	5.49 [1.13–26.62]	**0.035**

3C		
	Odds ratio [95% CI]	*p*-value
Moderate or severe disease at diagnosis	0.61 [0.15–2.47]	0.484
ESR at diagnosis	1.00 [0.97–1.03]	0.991
CRP at diagnosis	1.01 [0.99–1.03]	0.575
Corticosteroid or EEN treatment	1.47 [0.40–5.32]	0.560

## Discussion

In the majority of children with newly diagnosed moderate-to-severe CD, EEN or corticosteroid induction treatment bridging to AZA monotherapy according to ECCO-ESPGHAN guideline [5] is insufficient to reach the target of endoscopic remission. At 10 weeks, our rates of endoscopic remission without treatment escalation after induction with EEN or corticosteroid induction were lower than < 15% in both groups. This was an unexpected finding, and lower than reported in previously published studies [7, 18–21], particularly for the children treated with EEN [22]. However, patients in most of these studies had lower disease activity at baseline compared to the patients included in our study [7, 19–22]. This does not fully account for the study of Borrelli et al., which mostly included patients with moderate-to-severe CD [18]. In this study, endoscopic remission at 10 weeks was reported in 74% of patients treated with EEN, and in 33% of those treated with corticosteroids. There are several hypotheses to explain the low endoscopic remission rates we observed. First, a difference in definition of endoscopic remission was used. In this study endoscopic remission has been defined as SES-CD < 3 following the definition of mucosal healing [13]. This was stricter than a decrease in the endoscopic score of at least 50% relative to baseline used by Borrelli et al. [18, 23]. However, using the same definition, the number of patients with response in our cohort still would have been lower in the EEN group (31%), while in corticosteroid-treated patients achieving this endpoint was comparable to the Borrelli cohort (31%).

Second, we stopped EEN after median of 6 weeks whereas patients in the Borrelli cohort were treated for 10 weeks with EEN until endoscopy. This may suggest that children with moderate-to-severe CD may have higher endoscopic remission rates if they receive EEN treatment for a longer period (i.e., more than 6 weeks). This argument does not hold for the patients treated with corticosteroids. At 10 weeks, they were mostly still tapering corticosteroids. Third, in the 4 weeks after EEN was stopped, disease activity may have flared rapidly, including the rise of fcal levels, and remission may have been lost [24]. This last explanation implies patients may actually have achieved endoscopic remission after induction treatment with EEN, but endoscopic remission was not sustained after re-introduction of their normal diet.

Although there are several explanations for these unexpected results, our results show that when children are treated following the recently revised guidelines [5], treatment targets to induce and maintain remission are not achieved in the majority of the children with moderate-to-severe CD.

Surprisingly, the EEN-treated patients in our study decreased in linear growth during the year following EEN induction treatment [25, 26]. Despite that this could be partially caused by inherent variability of the length and weight measurements, we have proposed some possible explanations for this finding. In 19% of the patients, EEN induction treatment was prematurely stopped due to lack of response. Moreover, almost half of the patients received treatment escalation before week 14. This indicates that patients still had active disease, which may have a negative impact on linear growth [27]. Another factor which may have contributed to growth delay is the fact that 56% of EEN-treated patients received corticosteroids within 52 weeks [8]. This advocates, in line with previous studies, that a maximally effective therapy from diagnosis onwards is highly desired [4].

After induction of remission, AZA monotherapy was continued to maintain remission. It may take up to 16 weeks for AZA to be therapeutically effective [5]. Of the patients who used AZA and were in clinical remission without treatment escalation at 14 weeks, half of the patients were no longer in clinical remission without treatment escalation to IFX at 52 weeks despite adequate AZA metabolites in the majority of the patients. This suggests that after achieving disease remission by conventional treatment in children with moderate-to-severe CD, AZA monotherapy is regularly insufficient to maintain sustained disease remission.

Although in multivariable analysis corticosteroid-treated patients had a higher odds ratio to achieve treatment success at 14 weeks compared to EEN treatment, this association was not found in relation to treatment escalation with IFX after 52 weeks. In line with prior meta-analysis [6, 28], no significant differences were found in remission rates at 52 weeks between patients treated with EEN induction and corticosteroid induction treatment.

One of the limitations of this cohort is the small sample size. Indeed, the TISKids study was not powered to identify potential associations with treatment success within the conventional treatment group. Studies including translational research with sufficient power to discriminate patients at baseline to establish a personalized treatment strategy would be beneficial to discriminate patients at diagnosis. Moreover, patients were not randomized between the EEN and corticosteroid group, as this was up to the patient and parents, in accordance with the treating physician. Selection bias could have occurred. However, SES-CD scores were comparable between the two groups at baseline suggesting this was not the case.

One of the strengths of this study was the quantity and quality of the data collection. Even though EEN and oral corticosteroids are frequently used to induce remission, only few studies [18, 19, 29] report endoscopic findings following induction treatment. Probably because the bowel preparation and general anaesthesia or deep sedation prior to endoscopy are stressful for paediatric patients [30]. This may also explain why in our study not all children consented to the scheduled endoscopic evaluation which led to missing endoscopic data and a lower number of endoscopies.

In conclusion, children with moderate-to-severe newly diagnosed CD had low endoscopic remission rates without treatment escalation at 10 weeks after EEN or corticosteroid induction treatment bridging to AZA monotherapy. These results show, despite treatment according to recently updated guidelines, treatment targets often will not be achieved in children with moderate-to-severe CD. A personalized treatment strategy would be essential to achieve the target of endoscopic remission in these children. Our data suggest that patients with more severe inflammation are less likely to achieve clinical remission by EEN or corticosteroids, making them candidates for FL-IFX. Future studies are now ongoing [31] and are required to identify the specific patients who will benefit from EEN or corticosteroid induction treatment because patients should receive optimal effective treatment from diagnosis.

## Supplementary Information

Below is the link to the electronic supplementary material.

Supplementary file1 (DOCX 23 KB)

## Data Availability

No data are available. The data that support the findings of this study are available from the corresponding author, upon reasonable request.

## Abbreviations

AZA: Azathioprine
CD: Crohn’s disease
ECCO: European Crohn’s and Colitis Organisation
ESPGHAN: European Society for Paediatric Gastroenterology, Hepatology and Nutrition
EEN: Exclusive enteral nutrition
fcal: Faecal calprotectin
FL-IFX: First-line infliximab
MTX: Methotrexate
RBC: Red blood cell
IBD: Inflammatory bowel disease
IQR: Interquartile range
SES-CD: Simple Endoscopic Score for Crohn’s Disease
SDS: Standard deviation scores
TPMT: Thiopurine methyl transferase
wPCDAI: weighted Paediatric Crohn's Disease Activity Index
WHO: World Health Organization
6-TGN: 6-Thioguanine
