# Supplementary material for: Evaluation of exclusive enteral nutrition and corticosteroid induction treatment in new-onset moderate-to-severe luminal paediatric Crohn’s disease
Source: Eur J Pediatr. 2022 Jun 8;181(8):3055–65. doi: 10.1007/s00431-022-04496-7 (PMC9352605; doi:10.1007/s00431-022-04496-7)
Supplement: Supplementary file 1 — Supplementary file1 (DOCX 23 KB) [file 431_2022_4496_MOESM1_ESM.docx]

**Supplemental Tables**

|  | EEN (n=27) | Corticosteroids (n=20) | Total (n=47) |
| --- | --- | --- | --- |
| Erasmus Medical Center – Sophia Children’s Hospital, the Netherlands | 7/27 (26%) | 12/20 (60%) | 19/47 (40%) |
| Isala Hospital, the Netherlands | 3/27 (11%) | 2/20 (10%) | 5/47 (11%) |
| Maasstad Hospital, the Netherlands | 0/27 | 3/20 (15%) | 3/47 (6%) |
| Utrecht Medical Center-Wilhelmina Children’s Hospital, the Netherlands | 3/27 (11%) | 0/20 | 3/47 (6%) |
| Amphia Hospital, the Netherlands | 3/27 (11%) | 0/20 | 3/47 (6%) |
| Children’s Hospital Zagreb,  University of Zagreb Medical School, Croatia | 2/27 (7%) | 0/20 | 2/47 (4%) |
| Amsterdam University Medical Center-Emma Children’s Hospital, VU University, the Netherlands | 7/27 (26%) | 1/20 (5%) | 8/47 (17%) |
| University of Groningen, University Medical Center Groningen, the Netherlands | 0/27 | 1/20 (5%) | 1/47 (2%) |
| Catharina Hospital, the Netherlands | 1/27 (4%) | 0/20 | 1/47 (2%) |
| Jeroen Bosch Hospital, the Netherlands | 0/27 | 1/20 (5%) | 1/47 (2%) |
| Medical Spectrum Twente, the Netherlands | 1/27 (4%) | 0/20 | 1/47 (2%) |

**Supplemental Table 1: Overview of recruited patients per participating center.** EEN; Exclusive Enteral Nutrition

| Disease characteristics at week 10 | Received repeated endoscopy at week 10 (n=29) | Missing repeated endoscopy at week 10 (n=18) | P-value |
| --- | --- | --- | --- |
| Allocated treatment at baseline:  EEN  Corticosteroids | 16 (55%)  13 (45%) | 11 (61%)  7 (39%) | 0.689 |
| wPCDAI | 15 (0-22.5) | 25 (20-37.5) | **0.004** |
| CRP, mg/L | 13.0 (4.4-25.0) | 8.0 (4.0-23.5) | 0.775 |
| ESR, mm/hour | 17 (10-32) | 17 (6-33) | 0.225 |
| Leukocytes, 10^9^/L | 7.3 (5.9-9.0) | 7.9 (6.2-9.8) | 0.634 |
| Faecal calprotectin, µg/g | 543 (315-1060) | 698 (257-1188) | 0.604 |

**Supplemental Table 2: Disease characteristics at week 10 of patients received repeated endoscopy and missing repeated endoscopy.** Terminal ileum was not intubated in 2/13 (15%) of the patients treated with corticosteroids and in 5/16 (31%) of the patients treated with EEN (p=0.410). Data are n (%) or median (IQR)**.** EEN; Exclusive Enteral Nutrition, wPCDAI, weighted paediatric Crohn’s disease activity index; CRP, C-reactive protein; ESR, erythrocyte sedimentation rate.

|  | EEN (n=27) | Corticosteroids (n=20) |
| --- | --- | --- |
| Endoscopy  Week 10  Week 52 | 16 (59%)  6 (22%) | 13 (65%)  7 (35%) |
| Assessment of clinical remission  Week 6  Week 10  Week 14  Week 52 | 25 (93%)  23 (85%)  23 (85%)  23 (85%) | 18 (90%)  19 (95%)  19 (95%)  18 (90%) |
| Fcal levels  Week 10  Week 52 | 21 (78%)  17 (62%) | 16 (80%)  13 (65%) |

**Supplemental Table 3: Overview of available data per outcome measurement**. Not all data was complete for all patients at every visit. Analysis of each outcome measurement was performed on complete scores at each visit. EEN, Exclusive Enteral Nutrition**;** Fcal, faecal calprotectin
